# Post-transplant Lymphoproliferative Disorder Following Cardiac Transplantation

**DOI:** 10.3389/fcvm.2022.787975

**Published:** 2022-02-23

**Authors:** Rabea Asleh, Hilmi Alnsasra, Thomas M. Habermann, Alexandros Briasoulis, Sudhir S. Kushwaha

**Affiliations:** ^1^Department of Cardiovascular Diseases, Mayo Clinic, Rochester, MN, United States; ^2^Heart Institute, Hadassah University Medical Center, Faculty of Medicine, Hebrew University of Jerusalem, Jerusalem, Israel; ^3^Soroka University Medical Center, Ben Gurion University of the Negev, Beer Sheva, Israel; ^4^Division of Hematology, Department of Medicine, Mayo Clinic, Rochester, MN, United States; ^5^Division of Cardiovascular Disease, University of Iowa Hospitals and Clinics, Iowa City, IA, United States

**Keywords:** PTLD, heart transplantation, Epstein-Barr virus, immunosuppression, mTOR inhibitors, rituximab

## Abstract

Post-transplant lymphoproliferative disorder (PTLD) is a spectrum of lymphoid conditions frequently associated with the Epstein Barr Virus (EBV) and the use of potent immunosuppressive drugs after solid organ transplantation. PTLD remains a major cause of long-term morbidity and mortality following heart transplantation (HT). Epstein-Barr virus (EBV) is a key pathogenic driver in many PTLD cases. In the majority of PTLD cases, the proliferating immune cell is the B-cell, and the impaired T-cell immune surveillance against infected B cells in immunosuppressed transplant patients plays a key role in the pathogenesis of EBV-positive PTLD. Preventive screening strategies have been attempted for PTLD including limiting patient exposure to aggressive immunosuppressive regimens by tailoring or minimizing immunosuppression while preserving graft function, anti-viral prophylaxis, routine EBV monitoring, and avoidance of EBV seromismatch. Our group has also demonstrated that conversion from calcineurin inhibitor to the mammalian target of rapamycin (mTOR) inhibitor, sirolimus, as a primary immunosuppression was associated with a decreased risk of PTLD following HT. The main therapeutic measures consist of immunosuppression reduction, treatment with rituximab and use of immunochemotherapy regimens. The purpose of this article is to review the potential mechanisms underlying PTLD pathogenesis, discuss recent advances, and review potential therapeutic targets to decrease the burden of PTLD after HT.

## Introduction

*De novo* malignancy is an important cause of long-term morbidity and mortality in solid organ transplant (SOT) recipients. The incidence of *de novo* malignancy in adults has been reported to be ~20% after 10 years (1–5) and as high as 40–70% during a 20-year period of immunosuppression after transplantation (6–10). Heart transplant (HT) recipients are at particularly increased risk of developing malignancies after transplantation, which is increased up to 4-fold compared with renal transplant recipients (11–16). With the improvement of early survival following HT and the increasing number of older patients receiving HT (17), malignancy becomes relatively more important than other causes of morbidity and mortality post-transplant (18). Indeed, malignancy is the main cause of death at 5 years after HT (2).

Post-transplant lymphoproliferative disorder (PTLD) is a spectrum of lymphoid conditions associated with the use of potent immunosuppressive drugs after SOT or hematopoietic stem-cell (HSC) transplantation (19–21). PTLD is the second most frequent malignancy after skin cancers in HT recipients, representing up to 10% of *de novo* malignancies post-HT (14) contributing to the overall mortality of HT patients, with a 5-year overall survival rate in the pre-rituximab era of 20% (14). Most PTLD cases are B-cell neoplasms, and up to 35% occur within the 1st year following transplantation (early PTLD), with more than 50% of cases associated with Epstein–Barr virus (EBV) (22). This review describes updated information on PTLD, including diagnosis, prevalence and risk factors, highlights insights into the pathophysiology and examines treatment strategies and future directions of research to treat this devastating complication following HT.

## Epidemiology

Data from transplant registries during the past two decades have reported an increased incidence of PTLD (2, 23–25). Analysis of data from the U.S. Organ Procurement Transplant Network (OPTN)/United Network for Organ Sharing (UNOS) database on adult transplantation performed in the United States between 1999 and 2008 demonstrated that the incidence of PTLD was highest in lung recipients [5.72 per 1,000 person-years (PY)], intermediate in liver (2.44/1,000 PY) and heart recipients (2.24/1,000 PY), and lowest in kidney recipients (1.58/1,000 PY). In HT recipients, PTLD was previously reported as the third most common malignancy with incidence of 2.24/1,000 PY (23).

A recent national registry of adult and pediatric SOT recipients from the United States with data from 2005 to 2014 reported a 5-year cumulative incidence of PTLD ranging from 0.6 to 9% in adult transplant recipients and from 2 to 15.8% in pediatric transplant recipients, with the highest PTLD rates for intestine transplant (9%) (combined adult and pediatric data). The rates of adult PTLD for heart and kidney transplants were 0.9 and 0.6%, respectively. These rates were found to be lower in patients with EBV positive serology compared to those with negative serology (25). However, an earlier single-center analysis of biopsy-confirmed PTLD in 6,607 HSC and SOT recipients between 1989 and 2010 in Belgium, reported overall incidence of 2.12%, with the highest among HT recipients (5.0%) (26). Pediatric SOT recipients were noted to experience higher incidence of PTLD than adults, which can be attributed in large part to the development of primary EBV infection after transplantation (27, 28). Indeed, pediatric recipients were more commonly EBV mismatched than were adult recipients for all organ types (25).

The incidence of EBV-negative PTLD was reported to be increasing over time in a cohort of 176 SOT recipients (29). In contrast, EBV positive PTLD cases tend to occur early post- transplant whereas EBV-negative PTLD cases have a continued increase in incidence in each year (30). The data on incidence of PTLD after HT are derived from single-center and multicenter reports with incidence rates that range from 0.7 to 6.8% (Table 1) (5, 14, 15, 31–42). Kotton et al. (25) analyzed PLTD rates based on EBV serology and type of organ transplant, the overall rates of PTLD in adult HT subgroup was 0.9% in all serology, 2.1% in EBV-negative serology and 0.6% in EBV-positive serology. Similarly, in adult kidney transplant recipients, PTLD rates were 0.6% in all serology, 1.7% in EBV-negative serology, and 0.5% in EBV-positive serology.

**Table 1:** Published data on the incidence of post-transplant lymphoproliferative disorder in cardiac transplant recipients.

**References**	**Number of HT recipients**	**Number of PTLD cases**	**PTLD incidence**	**Follow-up time**
Couetil et al. (31)	275	2	0.7%	NA
Grattan et al. (32)	310	11	3.5%	NA
Swinnen et al. (33)	154	10	6.5%	NA
Armitage et al. (34)	439	15	3.4%	NA
Rinde-Hoffman et al. (35)	92	5	5.5%	NA
Chen et al. (36)	424	19	4.5%	0.5 years (median)
Mihalov et al. (37)	307	21	6.8%	NA
Hsu et al. (38)	156	4	2.6%	4.3 years (mean)
Yagdi et al. (15)	835	30	3.6%	9.6 years (median)
Crespo-Leiro et al. (14)	3,393	62	1.8%	5.2 years (median)
Fröhlich et al. (5)	255	18	7.0%	12.6 years (median).
Higgins et al. (39)	6,211	88	1.4%	5.5 years (median)
Rivinius et al. (40)	381	11	2.9%	9.7 years (mean)
Youn et al. (41)				NA
2000–2005	8,555	83	1.0%	
2006–2011	9,032	75	0.9%	
Asleh et al. (42)	523	24	4.6% (0.6 events per 100 person-years)	9 years (median)

*HT, heart transplant; PTLD, post-transplant lymphoproliferative disorder*.

## Risk Factors

The risk of PTLD is affected by the type of organ transplanted with the lowest risk observed in kidney transplant recipients compared to heart and lung transplant recipients (23, 26, 27). This may be, at least partially, explained by more intensive use of immunosuppression in recipients of thoracic organs. Although not fully understood, the increased incidence of PTLD after lung and intestinal transplantation may also be attributed to the large number of EBV-infected donor lymphocytes residing within these transplanted organs (27). In a previous study by Opelz and Döhler (43), the risk of lymphoma during the first post-transplant year was reported to be the highest for combined heart-lung recipients followed by lung, heart and kidney with the lowest risk. Moreover, the steepest long-term increase was noted for HT recipients.

The age of the transplant recipient is a factor affecting the risk of PTLD with greatest risk in both age extremes. SOT subjects aged < 10 and > 60 years were reported to be at increased risk of PTLD (43). Data from HT patients revealed that the HT recipient age was not associated with PTLD risk (14, 41, 42). One study found that recipient age < 18 years was strongly associated with increased risk of PTLD in HT recipients, which was independent of recipient EBV seronegative status (44).

Additionally, a higher incidence of PTLD has been reported in Caucasian SOT recipients (45, 46). However, this association has not been established in HT recipients in particular (44). SOT donor and recipient‘s genetic variation has been identified as a factor in the development of PTLD. SOT recipient positivity for HLA DR13 or B38, have been associated with higher risk of the PTLD (47), whereas donor haplotypes HLA-A1, HLA-B8, and HLA-DR3 were identified as protective factors (48). Furthermore, a higher degree of HLA mismatch was also associated with increased risk of PTLD (24, 47).

Donor to recipient EBV seromismatch (D+/R-), or (D-/R+) represents one of the strongest risk factors for PTLD development (44, 49–54). Moreover, the incidence of PTD post SOT has a bimodal curve, with an initial spike, mostly involving EBV-positive transplant recipients, during the first 12 months followed by a late spike, mostly involving EBV-negative recipients, 5–15 years after transplantation (26, 55, 56). Both EBV recipient serostatus (negative vs. positive) (41, 44) and EBV infection (42) were found to be strongly associated with increased risk of PTLD in HT patients.

An analysis of the SRTR National Registry Data in the United States was comprised of 112,756 kidney transplants (PTLD cases; 0.51%), 13,937 HT (1.0%), and 40,437 lung transplants (0.95%). EBV seronegative status at the time of transplant was associated with increased risk of PTLD with the highest risk in HT (44).

The risk of PTLD due to immunosuppression therapy is related to different immunosuppression approaches including T-cell depletion strategies. In SOT, the induction therapy with the monoclonal agent antibody, muromonab-CD3 (Orthoclone OKT3), when added to maintenance immunosuppression was found to be associated with higher risk of PTLD (33, 43). There are conflicting data regarding anti-thymocyte globulin (ATG) with some reports of increased risk of PTLD (43, 57) whereas others showed no increased risk of PTLD (58). Data from HT studies have reported an increased risk of PTLD associated with ATG (14, 41) but not with OKT (14). However, we have recently shown that the risk of PTLD was similar between HT recipients who received OKT3 and those received ATG induction therapy (42). Moreover, the associated OKT3 risk with PTLD in HT recipients has been shown to be dose-dependent (33).

Regarding maintenance immunosuppression, the contribution of each immunosuppressive agent is not clear, due to the frequent use of multiple agents in different doses and at different times post transplantation. Calcineurin inhibitors (CNIs; tacrolimus and cyclosporine) have been implicated as potential risk factors for PTLD following SOT (11, 24, 43). The multicenter Collaborative Transplant study (11) found that antithymocyte/antilymphocyte globulin or the monoclonal anti-T-cell antibody OKT3 use and use of a combination of cyclosporine and azathioprine wee independent risk factors of PTLD, but there was no increase in PTLD risk when cyclosporine was used alone.

Analysis of HT recipients from the SRTR National Registry Data in the United States reported that cyclosporine was associated with decreased risk of PTLD when compared to tacrolimus (44). However, data form HT patients showed that tacrolimus as an individual agent was not associated with PTLD risk (14). Although mycophenolate Mofetil (MMF) in standard immunosuppressive regimens after HT was found to be associated with a significantly lower risk of all *de novo* malignancies in general (59), it was not found to be associated with PTLD risk (14). When compared to MMF, azathioprine was not found to be associated with risk of PTLD post HT (41, 42, 44).

Sirolimus (SRL) has been shown to have antitumor and anti-EBV proliferation effects *in vivo* (60, 61). Data from kidney transplant patients suggests reduced risk of overall *de novo* malignancies (62, 63) and skin cancer (62–64). However, SRL use post transplantation was not found to be associated with decreased PTLD risk post kidney transplant (62).

A recent study from our group demonstrated that conversion to SRL as primary immunosuppression, with withdrawal of CNI therapy, was associated with a decreased risk of all *de novo* malignancies, PTLD, and subsequent primary occurrences of non-melanoma skin cancer after HT (42).

## Pathogenesis

**Figure 1:**
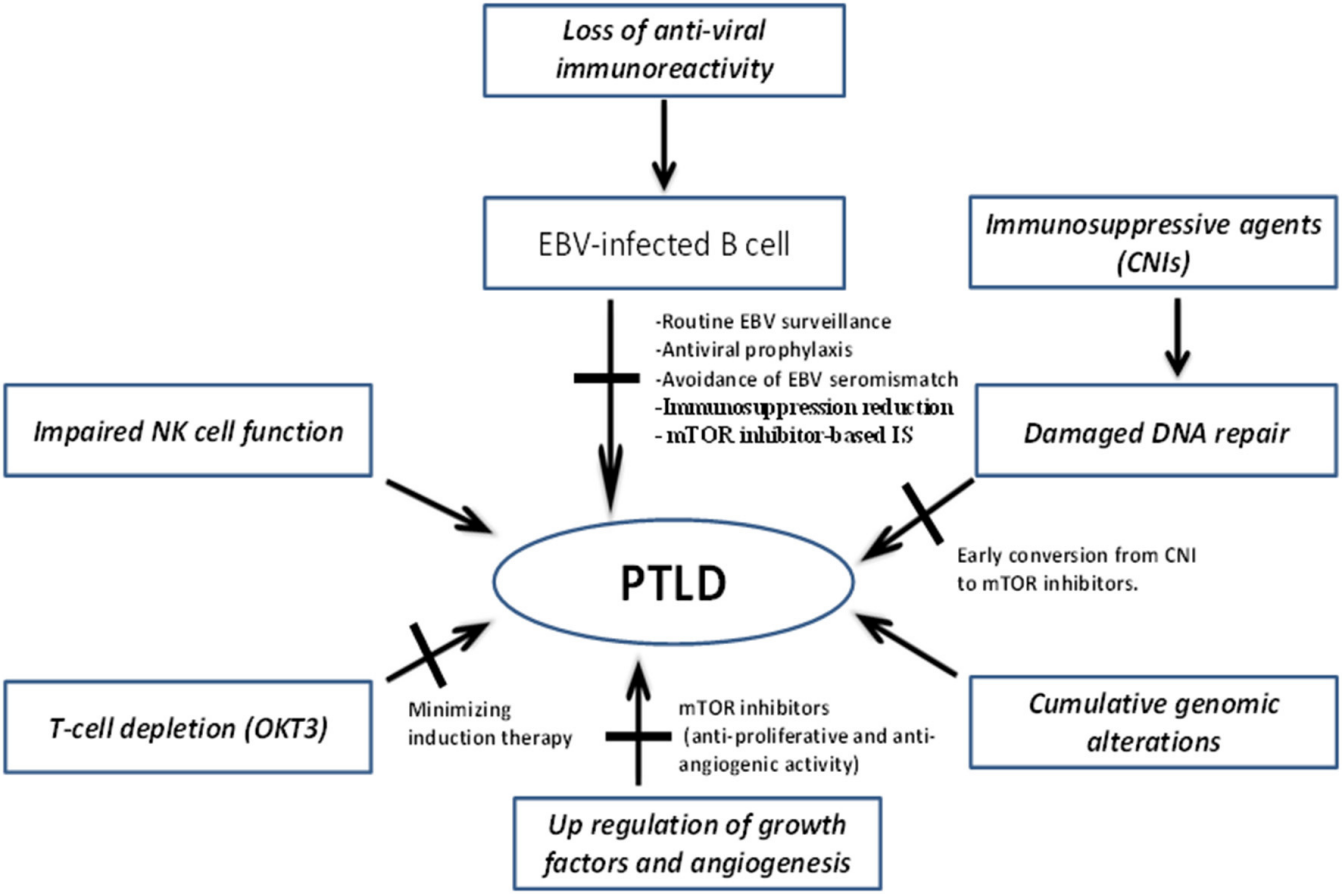
Mechanisms underlying pathogenesis of PTLD and potential targets to mitigate disease development and progression. CNIs, Calcineurin inhibitors; EBV, Epstein–Barr virus; IS, immunosuppression; mTOR, mammalian target of rapamycin; NK, natural killer; PTLD, post-transplant lymphoproliferative disorder.

The pathogenesis of PTLD involves one or more of the following mechanisms: (1) impaired immune surveillance of tumor cells due to immunosuppression; (2) decreased anti-viral immune activity and oncogenic effect of EBV; and (3) derangement of molecular signaling/DNA repair mechanisms by direct effect of immunosuppressive agents (Figure 1).

### The Role of EBV

The abnormal cell proliferation is driven in 50–80% of PTLD cases by EBV (49). The life cycle of EBV is initiated by an infection in immunocompetent hosts followed by lytic cycling and latency in the reticuloendothelial system. After that, EBV changes its viral gene program to express a type III gene latency program, characterized by expression of nine viral proteins including latent membrane protein 1 (LMP-1) and Epstein-Barr nuclear antigens (EBNAs-). To avoid host T lymphocyte recognition of these highly immunogenic latency III proteins, the virus transitions down further to a latency type II gene program, of which some genes provide surrogate co-stimulatory signals to host B cells to promote cell survival and differentiation. The resultant memory B cells have expressing EBV-encoded- RNA genes, concealing itself from host responses (65, 66).

In immunocompetent hosts, EBV-specific CD8+ effector and memory T cells are responsible for control of these EBV-infected B cells from abnormal and uncontrolled proliferation (67). This host T cell control of B cell proliferation is suppressed by immunosuppression (68). Therefore, Impaired T-cell immune surveillance against infected B cells in immunosuppressed transplant patients plays a key role in the pathogenesis of EBV-positive PTLD (69).

The pathogenesis of EBV-negative cases of PTLD is less clear. However, previous genomic studies demonstrated that EBV negative PTLD cases share genomic alterations seen in diffuse large B-cell Lymphoma (70–72) and T-cell lymphomas in immunocompetent patients (73). In contrast, EBV-positive PTLD cases have fewer such genomic abnormalities (74).

### The Role of Immunosuppression

The mechanisms of the increased risk for PTLD due to induction therapy with monoclonal induction antibodies are unclear. However, animal models showed that low T cell numbers at the time of transplantation from depleting antibodies increased the risk of PTLD (75). Moreover, impaired T-cell immune surveillance against EBV-infected B cells in immunosuppressed transplant patients plays a key role in the pathogenesis of EBV-positive PTLD (69). By expressing different latent antigens during B-cell development, EBV incorporates the normal B-cell program, thereby promoting proliferation and transformation of these cells. In normal circumstances, these antigens elicit a T-cell response that destroys the majority of EBV-infected B cells. This immunologic response is diminished in transplant recipients, hence increasing the risk of B-cell transformation and development of lymphomas.

CNIs exhibit pro-carcinogenic potential *via* inducing transforming growth factor-β production, which enhances tumor progression and angiogenesis, and inhibits DNA repair enzymes facilitating accumulation of mutations (76).

The decreased risk of overall post-transplant malignancies including PTLD in patients treated with SRL is related to its additive inhibitory effects on tumor growth, including antiproliferative and antiangiogenic activities beyond its immunosuppressive effect. The mammalian target of rapamycin (mTOR) pathway is a regulatory serine-threonine kinase, activated *via* the phosphatidylinositol-3-kinase (AKT) which has been implicated in progression of malignancies (77). SRL inhibits the (PI3K) signaling pathway contributing to the regulation of cell proliferation. SRL also inhibits transcription activator3 (STAT3) which mediates gene expression in cell growth and apoptosis and remains unregulated in many tumor types (78–80). Moreover, SRL exerts potent anti-angiogenic activity *in vitro* and *in vivo* in established tumors *via* inhibition of vascular endothelial growth factor (VEGF) production (81). Additionally, SRL has been shown to have anti-EBV proliferation effects *in vivo* (60, 61) and may avert growth of EBV-transformed B lymphocytes (82).

## Prevention

Prevention is an important measure, because the main risk factors for PTLD are EBV and the degree of immunosuppression. Strategies, such as limiting patient exposure to aggressive immunosuppressive regimens with rapid withdrawal or tapering of agents required for maintenance of graft function may decrease the incidence of PTLD. EBV monitoring has been incorporated into the routine evaluation of SOT patients. Avoidance of seropositive donors to seronegative recipients when multiple donor options are available is a measure that can further reduce the risk of PTLD.

While the degree of immunosuppression required and the timing of immunosuppression withdrawal differs, the consensus is that more aggressive withdrawal of immunosuppression to maintenance target concentrations is associated with lower incidence of PTLD. Among pediatric renal allograft recipients, the prevalence of PTLD has decreased with time, and this finding is attributed to policies of tapering CNIs to lower maintenance target trough concentration of 5–9 ng/mL (83). SOT recipients who are EBV-seronegative before transplant are commonly monitored for EBV viremia at regular intervals after transplant. Reduction of immunosuppression in patients with EBV viremia has been shown to reduce the incidence of early PTLD in pediatric SOT recipients (84, 85). The role of antiviral prophylaxis for PTLD prevention remains controversial for SOT recipients who are seronegative for EBV but receiving organs from seropositive donors. Retrospective observational studies have shown conflicting results (85–87), and a recent meta-analysis examining prophylactic or preemptive antiviral agents reported no significant effect on the incidence of PTLD across all types of organ transplants and age groups in high-risk EBV-naïve patients following SOT (88). A previous prospective study involving pediatric liver transplant showed that ganciclovir for 2 weeks immediately after transplant followed by 50 weeks of either acyclovir or placebo resulted in similar rates of PTLD (89). In the absence of convincing evidence, the use of antiviral agents as prophylaxis for PTLD prevention in EBV mismatched patients is not recommended (87).

The approach of preemptive treatment of PTLD at the time of viral reactivation with rituximab has been applied in allogeneic hematopoietic cell transplantation recipients and prevented PTLD without excess infections or mortality (90–92). In a series of 299 cardiac transplant patients, 31 had EBV reactivation and 6 had an EBV primary infection. Thirty-one patients had decreased immunosuppression and 15 had a single dose of rituximab at 375 mg/m^2^. All patients had a decrease in viral load. There was one possible PTLD and one death secondary to pulmonary PTLD. Unlike in PTLD complicating HSC transplants, the role of preemptive use of rituximab to prevent PTLD in SOT recipients is less clear. In the Swiss Transplant Cohort Study, no significant differences in the incidence of PTLD were found between SOT recipients receiving induction therapy with or without rituximab, although none of the patients (0/191) who received rituximab developed PTLD, while 57 of 4,574 (1.2%) patients without rituximab induction developed PTLD during follow-up (93). Therefore, further studies are warranted to determine the role of preemptive use of rituximab in preventing PTLD among SOT recipients.

In addition to tapering CNIs, our group has studied the effects of mammalian target of rapamycin (mTOR) antagonists on the incidence of post-transplant malignancies including PTLD among HT recipients (42). Sirolimus (SRL) and its derivative, everolimus, are mTOR inhibitors that suppress tumor growth in animal models (94) and have been successfully used in treating selective types of cancers (95). In HT recipients, studies assessing the effect of mTOR inhibition on the development of PTLD are lacking due to the relatively small pool of HT recipients treated with mTOR antagonists. A single-center retrospective analysis from our group showed that early conversion to a maintenance SRL-based immunosuppression, with complete withdrawal of CNIs, was associated with attenuation of cardiac allograft vasculopathy progression and improvement not only in cardiac outcomes but also in late survival after HT compared with continued CNI use over a mean follow-up of ~9 years (96). The improvement in late survival with SRL could not be entirely attributed to attenuation of CAV progression. Therefore, a subsequent analysis of malignancies from our center suggested that conversion to SRL was significantly associated with a decreased risk of PTLD (HR: 0.13; 95% CI: 0.03–0.59; *p* = 0.009) (42). The effects on PTLD were independent of EBV infection and type of induction therapy. The mechanisms behind which SRL confers protection against PTLD are not entirely clear. A previous study showed that the PI3K/Akt/mTOR pathway was constitutively active in EBV-positive B lymphomas from patients with PTLD, and that SRL combined with PI3K-δ inhibitor synergistically suppressed the proliferation of EBV-positive B lymphoma cells (97).

## Pathology

The World Health Organization (WHO) classified PTLD in four main categories based on morphologic, immunophenotypic, genetic, and clinical features: (i) Early lesions including plasmacytic hyperplasia and infectious mononucleosis-like PTLD, (ii) Polymorphic PTLD, (iii) Monomorphic PTLD, and (iv) Classic Hodgkin lymphoma-like PTLD (87). Initial management depends on the type of PTLD and immunosuppression reduction strategies.

## Treatment

The main strategies of PTLD treatment include reduction of immunosuppression, immunotherapy with the CD20 monoclonal antibody rituximab, chemotherapy, radiation therapy, adoptive immunotherapy with EBV-specific cytotoxic T lymphocytes, or a combination of these. The choice of strategy depends on the PTLD subtype, aggressiveness of PTLD, associated toxicities and the type of transplant. The main goals of therapy are eradication of PTLD and preservation of graft function. Not uncommonly, these goals are conflicting. Reduction of immunosuppression, which is commonly employed for PTLD eradication, increases the risk of graft rejection and it may not be feasible in single-organ transplants of vital organ (such as HT). In these cases, alternative therapies for PTLD must be used.

In accordance with the recommendations by the National Comprehensive Cancer Network (NCCN), the British Committee for Standards in Hematology, and the European Best Practice Guidelines for renal transplantation for most patients with early lesions, reduction of immunosuppression is the first step and additional agents are reserved for those who cannot tolerate reduction in immunosuppression and patient with residual disease (98–100). Data regarding the efficacy of reduction of immunosuppression are derived from observational studies in which patients also received other treatment strategies. Most early lesions either resolve completely or improve significantly within few weeks due to reduction of immunosuppression (101).

The optimal reduction of immunosuppression regimen depends on the histology, stage of PTLD, organ involvement, the presence of dual organ transplantation and the estimated risk related to graft loss or rejection. Steroid only reduction is not effective in most patients. Reduction by at least 50% the CNIs and discontinuation of other immunosuppressive drugs is recommended but not always feasible (53).

Polymorphic PTLD are defined as polyclonal or monoclonal lymphoid infiltrates that demonstrate evidence of malignant transformation but do not meet all of the criteria for one of the B cell or T/NK cell lymphomas (53, 102). Patients with polymorphic CD20-positive PTLD, receive rituximab in addition to reduced immunosuppression as an initial management strategy. Since polymorphic PTLD, by definition, consists of a mixture of monoclonal CD20-positive B-cell and polyclonal T-cell infiltrates, it is commonly treated with rituximab. Complete remission with rituximab monotherapy is relatively low in adult patients (<50%) (103–105) and identifies a population of patients that require additional chemotherapy. Pediatric patients have generally higher response rates to rituximab monotherapy (106, 107). For patients with monomorphic PTLD (those with monoclonal lymphoid proliferations that meet the criteria for one of the B cell or T/NK cell lymphomas), an approach including reduction in immunosuppression, rituximab and combination chemotherapy either concurrently or sequentially is indicated (22, 108). Patients with CD20-positive polymorphic PTLD with poor performance status or minimal symptoms may be candidates for rituximab alone. Combination chemotherapy with cyclophosphamide, doxorubicin, vincristine, and prednisone (CHOP), although not studied in randomized clinical trials vs. rituximab single therapy, achieved complete response in over 50% of cases (22, 105). The phase II sequential treatment of CD20-positive PTLD (PTLD-1) trial (22) involving 70 patients recruited from 2003 to 2007 has established sequential treatment with four cycles of weekly rituximab followed by four cycles of CHOP every 21 days (CHOP-21) as a standard of care in CD20-positive PTLD after SOL. Overall, 53 of 59 patients had a complete or partial response (90%) to sequential treatment, of which 40 (68%) were complete responses. The median survival using this regimen has significantly improved compared to the preceding rituximab monotherapy trials (6.6 years vs. 1.2–3.5 years, respectively) (103, 109, 110). Sequential therapy was also associated with less drug toxicity, particularly treatment-related mortality, as compared to the preceding retrospective case series of first-line chemotherapy in PTLD (13% vs. up to 31%, respectively) (22, 111). Importantly, the initial response to rituximab induction was found to be a prognostic factor for overall survival. This observation has led to a subsequent study demonstrating the feasibility, safety, and efficacy of treatment stratification into rituximab or rituximab plus CHOP consolidation according to response to rituximab induction (105). Consolidation therapy with rituximab only for patients who achieved a complete response after rituximab induction [88/126 patients (70%)] was safe (8% treatment-related mortality) and resulted in comparable median overall survival (6.6 years) compared to sequential therapy (PTLD-1) (22, 105). These findings demonstrate that rituximab without the need for chemotherapy is an appropriate therapeutic strategy when complete response is achieved after four cycles of rituximab induction in patients with CD20-positive PTLD complicating SOT. For patients not expressing CD20, chemotherapy without rituximab and surgery (for those with a localized disease) are indicated. T-cell lymphomas do not respond to rituximab and should be treated according to their pathology. Patients with classic Hodgkin lymphoma-like PTLD (the least frequent type of PTLD) should be treated according to the treatment standards of Hodgkin-lymphomas (112–114).

Radiation therapy can be used for patients with localized disease (115). In primary CNS PTLD, rituximab and high-dose methotrexate favorably impact survival (116) although CNS PTLD generally has a dismal prognosis. For patients with persistent disease despite reduction of immunosuppression and combined chemotherapy, adoptive immunotherapy with EBV-specific cytotoxic T lymphocytes (EBV-CTLs) can be used as a therapeutic option for high-risk rituximab-refractory cases, including promising results obtained among patients with CNS PTLD treated with EBV-CTLs (117). In an earlier report from 1994, 5 HSC transplant recipients with monoclonal EBV-induced PTLD achieved complete remission (CR) after infusion of lymphocytes (donor lymphocyte infusion) from their EBV-seropositive transplant donors (118). Subsequent small case series have confirmed that donor-derived EBV-CTLs induce clearance of viremia and persistent CR of EBV-associated lymphomas after HSC transplantation in 50–70% of patients (119, 120). In SOT patients, autologous EBV-CTLs have been shown to induce CR or transient partial remission (PR) of EBV-induced PTLD (121–124). However, autologous EBV-CTLs rarely result in clearance of EBV viremia (122, 124, 125). Additionally, their use is time-consuming and also limited in seronegative SOT recipients and in those treated with rituximab. Therefore, partially HLA-matched EBV-CTLs derived from healthy donors other than the transplant donor (third-party donors) have been investigated for treatment of refractory EBV-PTLD cases. Haque et al. (126) first reported the use of such cells in the treatment of 8 SOT recipients with EBV-induced PTLD in 2002. Subsequently, the same group reported on 31 SOT and 2 HCT recipients with EBV-induced PTLD, of whom 14 achieved CR and 3 achieved PR (127). Additional case series including small number of patients have used third-party EBV-CTLs to treat EBV-associated PTLD showing promising results and a favorable safety profile (128–130). A recent study by Prockop et al. (117) involving 46 recipients of allogeneic HSC transplant or SOT with established EBV-induced PTLD who had failed rituximab therapy has demonstrated that third-party EBV-CTLs that are partially HLA-matched and appropriately HLA restricted can induce durable CR or PR in a high proportion of patients without significant toxicity, graft injury, or graft-vs.-host disease (GvHD). Specifically, CR or sustained PR was achieved in 68% of HCT recipients and 54% of SOT recipients, and patients who achieved CR/PR or stable disease after cycle 1 had 1-year survival of 89 and 82%, respectively. These promising findings suggest that off-the-shelf EBV-CTLs can provide multiple immediately accessible options for potentially curative treatment of high-risk rituximab-refractory EBV-associated lymphomas complicating HSC transplantation or SOT.

## Prognosis

Old data from retrospective studies reported that mortality of monomorphic PTLD exceeds 80% and all PTLD types are associated with poor overall survival of <50% (131). However, most of these studies report outcomes before the rituximab era, which have improved survival. Predictors of worse outcomes include older age (>55 years), serum creatinine >1.5 mg/dL, elevated LDH, location of disease (central nervous system), and monomorphic or T cell (132), Eastern Cooperative Oncology Group (ECOG) performance status ≥2 and more than one site involvement (133). The introduction of rituximab has improved the outcomes of patients with CD20-positive PTLD. Moreover, response to rituximab induction remained a predictive marker for overall survival despite treatment stratification (22, 105). Patients who survive PTLD and undergo retransplantation, have excellent graft survival (134).

## Concluding Remarks

PTLD is a complication of chronic immunosuppression after HT, related to EBV activation resulting in proliferation of EBV-positive B cells in most cases. Prevention of PTLD is particularly important and this can be achieved with tapering immunosuppression, use of mTOR inhibitors in lieu of CNIs, routine surveillance of EBV viral loads, particularly in patients with EBV mismatch. Survival after PTLD has improved. The main therapeutic measures consist of immunosuppression reduction, treatment with rituximab in CD20-positive patients who achieve complete response to rituximab induction, and use of rituximab in combination with CHOP chemotherapy (R-CHOP) as consolidation in patients who do not achieve complete response to rituximab induction. Further studies are warranted to validate the role of mTOR antagonists, tailoring immunosuppression based on the risk of rejection, infection, and malignancies including PTLD. The use of novel types of chemotherapy and immunotherapy in PTLD is under investigation.

## Author Contributions

All authors listed have made a substantial, direct, and intellectual contribution to the work and approved it for publication.

## Conflict of Interest

The authors declare that the research was conducted in the absence of any commercial or financial relationships that could be construed as a potential conflict of interest.

## Publisher's Note

All claims expressed in this article are solely those of the authors and do not necessarily represent those of their affiliated organizations, or those of the publisher, the editors and the reviewers. Any product that may be evaluated in this article, or claim that may be made by its manufacturer, is not guaranteed or endorsed by the publisher.

## Abbreviations

CNI: calcineurin inhibitor
CR: complete remission
EBV: Epstein-Barr virus
HSC: hematopoietic stem cell
HT: heart transplantation
mTOR: mammalian target of rapamycin
NK: natural killer
OKT3: muromonab-CD3
PR: partial remission
PTLD: post-transplant lymphoproliferative disorder
SOT: solid organ transplant
SRL: sirolimus.
